# The IL-33/ST2 axis affects tumor growth by regulating mitophagy in macrophages and reprogramming their polarization

**DOI:** 10.20892/j.issn.2095-3941.2020.0211

**Published:** 2021-02-15

**Authors:** Huadan Xu, Dong Li, Jiaoyan Ma, Yuanxin Zhao, Long Xu, Rui Tian, Yanan Liu, Liankun Sun, Jing Su

**Affiliations:** 1 Key Laboratory of Pathobiology, Ministry of Education, Department of Pathophysiology, College of Basic Medical Sciences, Jilin University, Changchun 130000, China; 2 Department of Hepatology, The First Hospital of Jilin University, Changchun 130000, China; 3 Department of Immunology, College of Basic Medical Sciences, Jilin University, Changchun 130000, China

**Keywords:** IL-33/ST2, macrophage polarization, mitophagy, glucose metabolism, tumor microenvironment

## Abstract

**Objective:**

Macrophages are a major component of the tumor microenvironment. M1 macrophages secrete pro-inflammatory factors that inhibit tumor growth and development, whereas tumor-associated macrophages (TAMs) mainly exhibit an M2 phenotype. Our previous studies have shown that the interleukin-33/ST2 (IL-33/ST2) axis is essential for activation of the M1 phenotype. This study investigates the role of the IL-33/ST2 axis in TAMs, its effects on tumor growth, and whether it participates in the mutual conversion between the M1 and M2 phenotypes.

**Methods:**

Bone marrow-derived macrophages were extracted from wildtype, ST2 knockout (ST2^−/−^), and Il33-overexpressing mice and differentiated with IL-4. The mitochondrial and lysosomal number and location, and the expression of related proteins were used to analyze mitophagy. Oxygen consumption rates and glucose and lactate levels were measured to reveal metabolic changes.

**Results:**

The IL-33/ST2 axis was demonstrated to play an important role in the metabolic conversion of macrophages from OXPHOS to glycolysis by altering mitophagy levels. The IL-33/ST2 axis promoted enhanced cell oxidative phosphorylation, thereby further increasing M2 polarization gene expression and ultimately promoting tumor growth (*P* < 0.05) (**Figure 4**). This metabolic shift was not due to mitochondrial damage, because the mitochondrial membrane potential was not significantly altered by IL-4 stimulation or ST2 knockout; however, it might be associated with the mTOR activity.

**Conclusions:**

These results clarify the interaction between the IL-33/ST2 pathway and macrophage polarization, and may pave the way to the development of new cancer immunotherapies targeting the IL-33/ST2 axis.

## Introduction

Many studies have indicated that the presence of macrophages in the tumor microenvironment is associated with enhanced tumor progression: macrophages have been shown to promote tumor growth, spreading, angiogenesis, and immunosuppression^1^. In most tumor microenvironments, macrophages are the predominant matrix component, composing as much as 50% of the entire microenvironment^2–4^. Therefore, the macrophagic mechanism of action is receiving increasing attention, and tumor-associated macrophages (TAMs) have been considered as potential therapeutic targets^1,5^. As observed in murine and human tumors, TAMs are poor producers of nitric oxide, and they express low levels of inflammatory cytokines such as interleukin-1 (IL-1), tumor necrosis factor (TNF), and IL-6^6^. In addition, TAMs exhibit defective nuclear factor-κB (NF-κB) pathway activation in response to lipopolysaccharides (LPS) and TNF stimulation^7–9^. This phenotype is similar to that of M2 macrophages.

Macrophagic plasticity affects tumor growth and progression, because different metabolic characteristics shape different macrophagic phenotypes^2^. Metabolism regulates the activation and polarization of macrophages. M1 macrophages exhibit metabolic characteristics dominated by aerobic glycolysis, similarly to the Warburg effect in tumor cells. Microbes and other environmental stimuli require rapid responses^7^; thus, M1 macrophages increase autophagy^10,11^ as an adaptive mechanism to such stimuli. In contrast, M2 macrophages use oxidative phosphorylation (OXPHOS) as the main metabolic method, and autophagy is inhibited or blocked^10,12,13^. Therefore, understanding the distinct aspects of mitochondrial metabolism (aerobic glycolysis and OXPHOS) of macrophages under physiological and pathological conditions might provide new targets for anti-tumor therapies^14^. Mammalian target of rapamycin (mTOR) is a serine/threonine kinase that regulates cell metabolism and is involved in macrophage activation^15,16^. In M2 macrophages, mTOR complex 1 (mTORC1) is activated in response to IL-4 stimulation, thereby promoting the formation of adaptive metabolic patterns and ultimately macrophage activation^16–18^. Autophagy occurs downstream of the mTOR signaling pathway and is inhibited by the phosphorylation of serine 757 of ULK1^15^. Therefore, the main purpose of this study was to elucidate the mechanisms of autophagy and metabolism, which affect the adaptive activation and polarization status of macrophages.

IL-33 is a pleiotropic cytokine with important roles at all stages of the macrophage-related immune response, including initiation^19^, maintenance^20–22^, and the final resolution stage^23,24^. IL-33 promotes both M1 and M2 macrophage polarization^19,25,26^; however the underlying mechanism remains unclear. Our previous studies have shown that mitochondrial metabolism is a core player in the macrophage polarization process. The IL-33/ST2 axis plays an important role in the metabolic reprogramming of M1 macrophages, through regulating the level of peroxisome proliferator-activated receptor gamma coactivator 1-alpha (PGC1α), which is associated with mitochondrial biosynthesis^27^. However, the role of IL-33 in regulating M2 macrophage polarization remains elusive^20,25,28^.

In this study, the importance of the IL-33/ST2 pathway in IL-4-stimulated macrophage metabolic reprogramming is illustrated. These results might aid in understanding of how macrophages initiate stimulation-induced responses.

## Materials and methods

### Animal experiments

Specific-pathogen-free 6–9-week-old male BALB/c mice were purchased from Beijing Vital River Laboratory Animal Technology Co., Ltd. (Beijing, China) and housed in specific-pathogen-free conditions at Jilin University (15). ST2^−/−^ mice were kindly provided by Prof. Weihua Xiao from the University of Science and Technology of China (Hefei, China), and Il33 transgenic mice were kindly provided by Prof. Ying Sun from Capital Medical University (Beijing, China). Both strains had a BALB/c background^23^. Melanoma B16 cells (RRID: CVCL_F936) (5.0 × 10^6^) were injected subcutaneously into the backs of mice. The tumor volumes (volume = 1/2 × long diameter × short diameter^2^) and the weights of the mice were measured every 7 days. On the 28th day, the mice were sacrificed. All animal experiments were performed in accordance with the National Guidelines for Experimental Animal Welfare, with approval from the Animal Welfare and Research Ethics Committee at Jilin University (Approval No. 2019-40) (Changchun, China).

### Cell culture

Primary bone-marrow derived macrophages (BMDMs) were generated and cultured as previously described^27^. IL-4 was purchased from BioLegend (San Diego CA, US). All other tissue culture reagents were purchased from Sigma-Aldrich (St. Louis, MO, US) unless otherwise stated.

### Quantitative polymerase chain reaction (qPCR)

Total RNA was extracted from BMDMs as previously described^27^. Genomic DNA digestion and reverse transcription were performed according to the manufacturer’s instructions. The primers used were as follows:

*Mrc1*F: 5′-CTCTGTTCAGCTATTGGACGCCG-3′R: 5′-TGGCACTCCCAAACATAATTTGA-3′;*Arg1*F: 5′-CTCCAAGCCAAAGTCCTTAGAG-3′R: 5-AGGAGCTGTCATTAGGGACA-3′;*Ym1*F: 5′-ATGAAGCATTGAATGGTCTGAAAG-3′R: 5′-TGAATATCTGACGGTTCTGAGGAG-3′;*Actb*F: 5′-CGTTGACATCCGTAAAGACC-3′R: 5′-AACAGTCCGCCTAGAAGCAC-3′.

### Measuring mitochondrial ROS production

The mitochondrial ROS were measured with a mitochondrial active oxygen kit (Thermo Fisher Scientific) according to the manufacturer’s instructions, by flow cytometry with a BD Accuri C6 instrument (BD Biosciences, Franklin Lakes, NJ, US).

### Detecting glucose uptake and lactic acid production

Culture medium was collected for glucose and lactate measurements with glucose and lactate assay kits (Beyotime, Haimen, Jiangsu, China), as previously described^29^.

### Analysis of the oxygen consumption rate (OCR) and extracellular acidification rate (ECAR)

The OCR and ECAR were measured with Mito-Xpress and pH-Xtra fluorescent probes (Luxcel Bioscience, Cork, Ireland), as previously described^27^.

### Measuring intracellular ATP production

Intracellular ATP production was measured with an Enhanced ATP Test Kit (Beyotime), as previously described^27^.

### Measuring mitochondrial membrane potential

MMP in BMDMs was determined with the JC-1 probe from the Membrane Potential Assay Kit (Beyotime) and then analyzed with FlowJo software (version 10.0.7; FlowJo, LLC, OR, US), as previously described^29^.

### Immunofluorescence

According to the manufacturer’s instructions, MitoTracker RED and LysoTracker GREEN (Thermo Fisher Scientific) were used to monitor the content and location of mitochondria and lysosomes in living cells.

The cells were incubated with primary antibodies against Parkin (Proteintech, Wuhan, Hubei, China) and VDAC1 (Santa Cruz Biotechnology, Dallas, US), then incubated with fluorescent secondary antibody (Proteintech, Wuhan, Hubei, China). Cells were imaged with a fluorescence microscope (ECHO, San Diego, US).

### Western blot analysis

Antibodies against VDAC1, Cytc, Hsp60, Parkin, PINK1, p62, p70s6k, P-p70s6k, and LC3 I/II were obtained from Santa Cruz Biotechnology (Dallas, TX, US); antibody to β-actin and all secondary antibodies were obtained from Proteintech (Wuhan, Hubei, China). The specific assay procedures were as previously described^27^.

### Gene Expression Profiling Interactive Analysis (GEPIA)

GEPIA is a newly developed interactive web server for analyzing the RNA sequencing expression data of 9,736 tumor and 8,587 normal samples from the TCGA and GTEx projects. GEPIA performs survival analysis on the basis of gene expression levels.

### Statistical analysis

Data are expressed as means ± standard error of the mean (SEM). The statistical significance between 2 groups was analyzed with one-way ANOVA followed by Student’s t-test in Prism software (GraphPad Software, La Jolla, CA, US). * represents *P* < 0.05 and was considered statistically significant. All experiments were repeated at least 3 times.

## Results

### ST2^−/−^ decreases expression of M2 marker genes in macrophages and increases glucose uptake and lactic acid production

**Figure 1:**
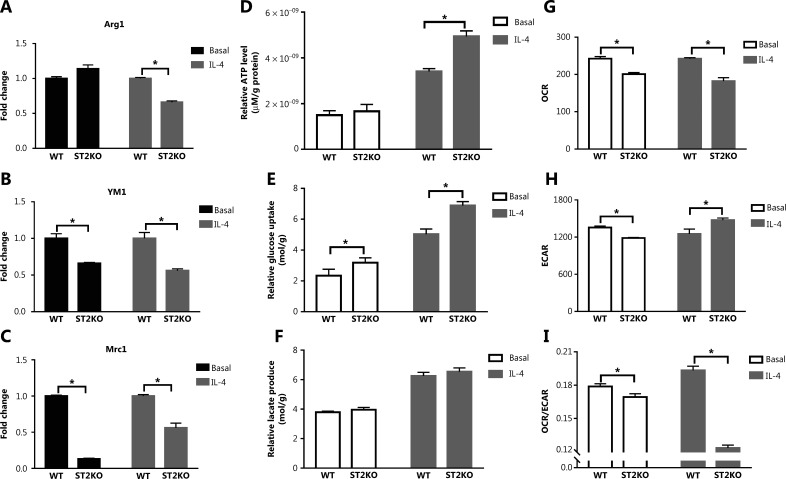
ST2−/− reduces M2 marker gene expression in macrophages, and increases glucose uptake and lactic acid production. BMDMs were stimulated with IL-4 (25 ng/mL) for 24 h. The expression of Arg1 (A), Ym1 (B) and Mrc1 (C) was detected by qPCR. The extracellular relative ATP level (D), relative glucose uptake (E), and relative lactic acid production (F) were measured after the above treatment. The extracellular oxygen consumption rate (OCR) (G) was measured immediately with an oxygen-sensitive probe. The extracellular acidification rate (ECAR) (H) after incubation at 37 °C for 3 h. Quantitative graph of the ratio of OCR to ECAR (I). Vertical bars = SEM (*n* = 3). * *P* < 0.05 ST2KO *vs.* WT at the same treatment.

To examine the role of the IL-33/ST2 axis in macrophages, we investigated the expression of M2 marker genes and compared the metabolic characteristics of BMDMs at baseline *vs.* after IL-4 stimulation. ST2^−/−^ BMDMs showed decreased expression of M2 marker genes under IL-4 stimulation (*P* < 0.05) (**Figure 1A–1C**). Furthermore, ST2^−/−^ BMDMs showed increased ATP levels (*P* < 0.05) (**Figure 1D**) and glucose consumption (*P* < 0.05) (**Figure 1E**) under IL-4 stimulation as compared with basal conditions. In the ST2^−/−^ compared with the wildtype (WT), the production of lactate (**Figure 1F**) was not significantly different. Therefore, we detected OCR and ECAR through a sensitive fluorescence real-time monitoring method to comprehensively evaluate the changes in cell metabolism. The OCR decreased (*P* < 0.05) (**Figure 1G**), and the ECAR increased under stimulation by IL-4 (*P* < 0.05) (**Figure 1H**). The OCR to ECAR ratio represents the proportion of OXPHOS and glycolysis. A decrease in this ratio was observed in ST2 deficient BMDMs, thus indicating that, by hindering the IL-33/ST2 signaling pathway after IL-4 stimulation, these macrophages undergo aerobic glycolysis similar to the Warburg effect.

### Fewer mitochondria in ST2-deleted macrophages are associated with increased mitochondrial autophagy

**Figure 2:**
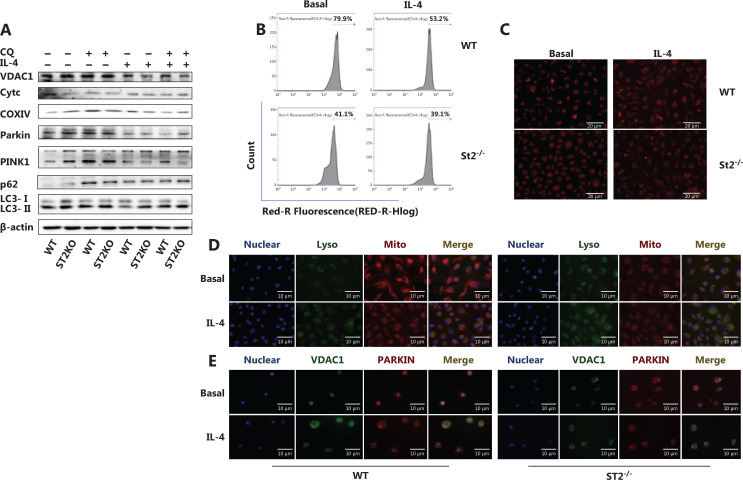
A decreased mitochondrial number in ST2^−/−^ macrophages is associated with increased mitochondrial autophagy. BMDMs were cultured as described above with/without IL-4 (25 ng/mL) and CQ (10 μM) for 24 h. Analysis of VDAC1, Cytc, COXIV, Parkin, PINK1, p62, LC3II and I by Western blot (A), and incubation with MitoTracker probe at 37 °C for half an hour, and flow cytometry (B) and fluorescence staining (C) (scale bar: 20 μm) to detect the number of labeled mitochondria are shown. BMDMs were cultured as described above. Fluorescence staining was used to detect the localization of mitochondria and lysosomes (scale bar: 10 μm) (D). Different fluorescent tags were used to detect the localization of VDAC1 and Parkin (scale bar: 10 μm) (E). Data are representative of 3 experiments.

Metabolic changes in macrophages are closely associated with mitochondrial number and function. Mitochondria-related indicators were examined to investigate the mechanism underlying the IL-33/ST2 axis in macrophage metabolism. The number of mitochondria was significantly lower in ST2^−/−^ than WT BMDMs (**Figure 2B and 2C**). Under IL-4 stimulation, the expression of the outer mitochondrial membrane protein voltage-dependent anion channel 1 (VDAC1), membrane gap protein cytochrome c, and inner membrane protein cytochrome c oxidase subunit IV (COXIV) was lower than that in WT macrophages (**Figure 2A**). In addition, the expression of microtubule associated protein light chain 3, MAP-LC3 (LC3II/LC3I), PTEN-induced putative kinase 1 (PINK1) (full length), and Parkin was higher, and p62 was lower, than that in WT macrophages (**Figure 2A**). After the addition of chloroquine (CQ) to inhibit lysosomal functions, the expression trends for VDAC1, Cytochrome C, COXIV, LC3II, LC3I, and full length PINK1 were reversed (**Figure 2A**).

To further determine the occurrence of mitophagy, we tagged mitochondria, lysosomes, VDAC1, and Parkin with immunofluorescent labels. Compared with the WT macrophages, ST2-deleted BMDMs presented greater mitochondrial and lysosomal fusion (**Figure 2D**) and colocalization of VDAC1 and Parkin (**Figure 2E**). These results suggest that the increase in mitophagy may be the reason for the metabolic changes observed in ST2^−/−^ BMDMs.

### Mitochondrial autophagy defects increase the expression of M2 marker genes, and decrease glucose uptake and lactic acid production in ST2^−/−^ macrophages stimulated with IL-4

**Figure 3:**
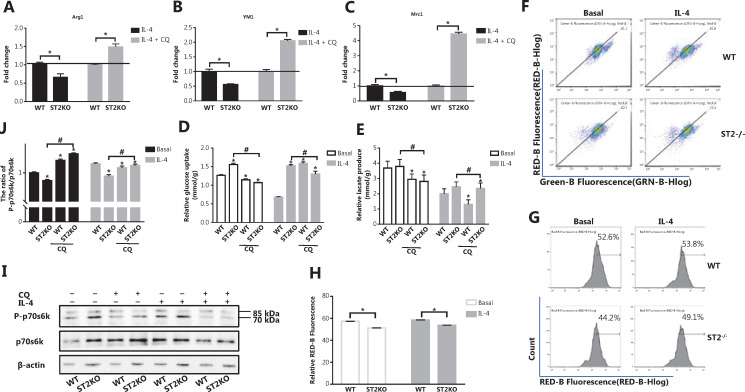
After CQ treatment, the expression of M2 marker genes in ST2^−/−^ BMDMs stimulated by IL-4 increases, and metabolic changes in ST2^−/−^ macrophages are not associated with mitochondrial damage. BMDMs were cultured as described in **Figure 2**. Arg1 (A), Ym1 (B), and Mrc1 (C) expression was assessed by qPCR, * *P* < 0.05 ST2KO *vs.* WT at the same treatment. Glucose uptake (D) and lactic acid production (E) were detected with specific absorbance analysis, * *P* < 0.05 *vs.* WT at the basal level, # *P* < 0.05 treatment with CQ* vs.* no CQ when ST2 was knocked out. BMDMs were cultured as described in **Figure 1**, and detection of JC-1 stained mitochondrial membrane potential (F) and MitoSOX stained mitochondrial ROS (G) by flow cytometry (H) were performed, on the basis of the statistical results represented in **Figure 3G**. BMDMs were cultured as described in **Figure 2**, and p70s6k and P-p70s6k were analyzed by Western blot (I). Quantitative graph of the intensity of P-p70s6k to p70s6k protein in (I) (J). Vertical bars = SEM (*n* = 3). * *P* < 0.05 *vs.* WT in basal or IL-4 treated conditions, # *P* < 0.05 treatment with CQ *vs.* no CQ when ST2 was knocked out.

Having demonstrated the reason for the metabolic changes in ST2^−/−^, we next examined related indicators after blocking mitophagy. ST2^−/−^ BMDMs showed lower M2 marker gene expression than that in WT macrophages after stimulation with IL-4 (**Figure 1A–1C**). However, these gene expression patterns were reversed after CQ treatment (*P* < 0.05) (**Figure 3A–3C**). The glucose consumption (*P* < 0.05) (**Figure 3D**) and lactic acid production (*P* < 0.05) (**Figure 3E**) in macrophages extracted from ST2-deficient mice were lower after CQ treatment compared with those in the untreated controls under basal and IL-4 stimulation conditions. This result indicates that increased mitophagy may be the reason for the metabolic changes observed in ST2^−/−^ macrophages and the eventual weakening of the M2 polarization tendency of macrophages.

### Metabolic changes caused by ST2^−/−^ are not associated with mitochondrial damage

In general, the disruption of mitochondrial membrane potential (MMP) is a direct cause of mitophagy. However, the absence of ST2 did not decrease the MMP (**Figure 3F**), and mitochondrial ROS levels did not increase but instead showed a relative decrease (**Figure 3G and 3H**) under both basal and IL-4 stimulation conditions. This result demonstrates that the impairment of the IL-33/ST2 axis in macrophages did not cause mitochondrial damage. Interestingly, the ratio of phosphorylated p70s6k to p70s6k was lower in ST2^−/−^ macrophages, but higher in the CQ-treated group, than in the corresponding untreated group (*P* < 0.05) (**Figure 3I and 3J**). This result indicated that mTOR activity was decreased in ST2^−/−^ macrophages and may be responsible for the increase in mitophagy.

### IL-33 overexpression increases M2 marker gene expression in macrophages and decreases glucose uptake and lactic acid production

**Figure 4:**
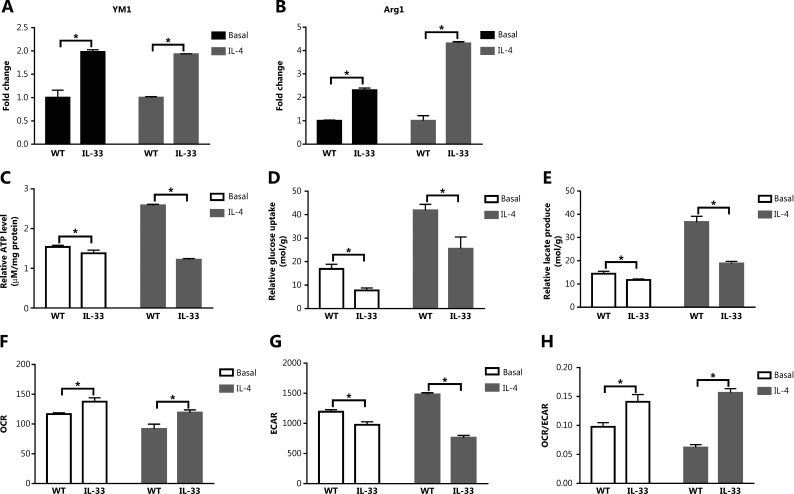
IL-33 overexpression increases M2 marker gene expression in macrophages, and decreases glucose uptake and lactic acid production. BMDMs from BALB/c or Il33 overexpressing (Il33Tg) mice were cultured in fresh medium or stimulated with IL-4 (25 ng/mL) for 24 h. The expression of Ym1 (A) and Arg1 (B) was evaluated with qPCR. Extracellular relative ATP levels (C), relative glucose uptake (D), and relative lactic acid production (E). An oxygen-sensitive probe was used to immediately measure the extracellular oxygen consumption rate (OCR) (F). The extracellular acidification rate (ECAR) was measured by incubation at 37 °C for 3 h (G). A quantitative graph of the ratio of OCR to ECAR (H). Vertical bars = SEM (*n* = 3). * *P* < 0.05 Il33Tg *vs.* WT under the same treatment.

To further elucidate the role of the IL-33/ST2 axis in macrophages, we investigated the expression levels of M2 marker genes and the metabolic characteristics of IL-33-overexpressing BMDMs under basal and IL-4 stimulation conditions. In contrast to ST2^−/−^ BMDMs, the IL-33-overexpressing BMDMs showed greater expression of M2 marker genes (Arg1 and YM1) than that in WT BMDMs under basal and IL-4 stimulation conditions (*P* < 0.05) (**Figure 4A and 4B**). Furthermore, IL-33 overexpression increased the ATP levels in BMDMs (*P* < 0.05) (**Figure 4C**) but decreased glucose consumption (*P* < 0.05) (**Figure 4D**) and lactic acid production (*P* < 0.05) (**Figure 4E**). Because the OCR increased in IL-33 overexpressing BMDMs while the ECAR decreased, these macrophages presented an increased OCR to ECAR ratio (*P* < 0.05) (**Figure 4F**), whereas the ECAR showed a decreasing trend (*P* < 0.05) (**Figure 4G**), and the ratio of OCR to ECAR increased (*P* < 0.05) (**Figure 4H**). These results indicated that enhanced IL-33/ST2 signaling promotes OXPHOS in macrophages, but decreases glucose uptake and lactic acid production.

### IL-33-overexpressing macrophages present increased mitochondrial numbers and consequently decreased mitochondrial autophagy

**Figure 5:**
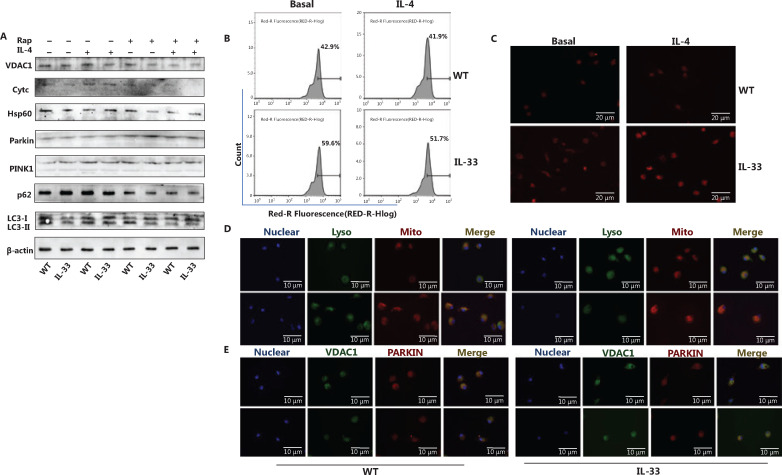
IL-33 overexpression increases the number of mitochondria and decreases mitochondrial autophagy. BMDMs from BALB/c or Il33Tg mice were cultured in fresh medium, with or without IL-4 (25 ng/mL) and rapamycin (Rap) (10 μM). After 24 h of stimulation, the expression of VDAC1, Cytc, Hsp60, Parkin, PINK1, p62, LC3II and I was analyzed by Western blot (A). BMDMs were cultured as described in **Figure 6**. The number of labeled mitochondria was measured by flow cytometry (B) and fluorescence staining (C) (scale bar: 20 μm). BMDMs were cultured as described in **Figure 6**. LysoTracker was used to label lysosomes, and MitoTracker was used to label mitochondria to detect the localization of mitochondria and lysosomes (scale bar: 10 μm) (D). After BMDMs were treated as described above and incubated overnight at 4 °C, they were incubated with secondary antibodies with different fluorescent tags for 1 h to detect the localization of VDAC1 and Parkin (scale bar: 10 μm) (E). Data are representative of 3 experiments.

The number of mitochondria was significantly higher in IL-33-overexpressing BMDMs than WT BMDMs (**Figure 5B and 5C**). After IL-4 stimulation, the expression of the mitochondrial inner membrane protein Hsp60 was greater in IL-33 overexpressing BMDMs than in WT macrophages (**Figure 5A**). IL-33-overexpressing BMDMs, compared with WT BMDMs, also showed lower expression of LC3II/LC3I, PINK1 (full length), and Parkin, and higher expression of p62 (**Figure 5A**). Rapamycin (Rap) was used to inhibit mTOR activity, thus reversing the aforementioned protein expression trends (**Figure 5A**). In addition, mitochondrial and lysosomal fusion and colocalization of VDAC1 and Parkin were lower in IL-33-overexpressing BMDMs than WT macrophages (**Figure 5D and 5E**). These results suggested that inhibition of mitophagy may be the reason for the metabolic changes observed in IL-33 overexpressing macrophages.

### Rapamycin increases glucose uptake and lactate production in IL-33-overexpressing BMDMs after IL-4 stimulation

**Figure 6:**
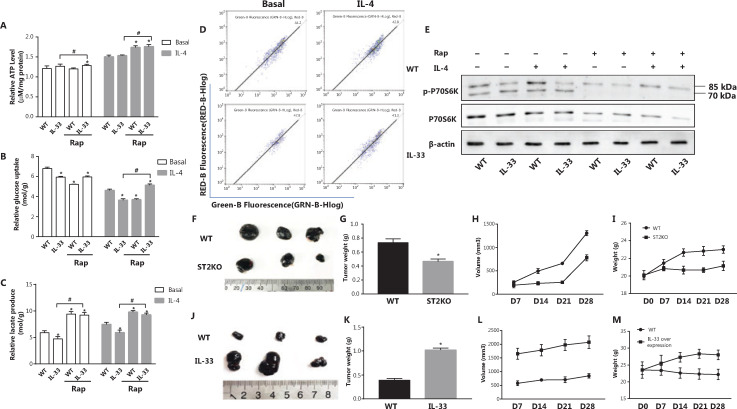
Rap increases IL-4 stimulated glucose uptake and lactate production of Il33Tg BMDMs, and IL-33/ST2 signals in macrophages promote tumor growth in mice. BMDMs were cultured as described in **Figure 1**. Relative ATP levels (A), glucose uptake (B) and relative lactic acid production (C). * *P* < 0.05 *vs.* WT in basal or IL-4 treated conditions, # *P* < 0.05 treatment with Rap *vs.* no Rap when IL-33 was overexpressed. BMDMs from BALB/c or Il33Tg mice were cultured with or without IL-4 (25 ng/mL). After 24 h of stimulation, the MMPs stained with JC-1 were detected by flow cytometry (D). After the same treatment as in (A), p70s6k and P-p70s6k were analyzed by Western blot (E). Wild-type and ST2KO mice were inoculated with B16 cells on the back (6 mice per group; representative results are shown). Tumor pictures (F) were taken, and average tumor weights (G) were determined on the 28th day for tumor-bearing mice; average tumor volume (H) was measured every 7 days. Average mouse body weight (I) was determined. B16 cells were inoculated on the backs of wild-type and IL-33 overexpressing mice, and tumor pictures were taken on day 28, when tumor-bearing mice were sacrificed (J). The average tumor weight (K), average tumor volume (L) and average mouse weight (M) were measured every 7 days. Vertical bars = SEM (*n* = 3). Data are representative of 3 experiments.

According to the results presented above, IL-33 overexpression led to greater ATP levels (*P* < 0.05) (**Figure 6A**), glucose consumption (*P* < 0.05) (**Figure 6B**), and lactic acid production (*P* < 0.05) (**Figure 6C**) than those observed in the BMDM group stimulated with IL-4 but not treated with Rap. This result indicated that inhibition of mitophagy may be a reason for the metabolic shift of IL-33-overexpressing macrophages to OXPHOS. Furthermore, overexpression of IL-33 did not cause a significant MMP change relative to that of BMDMs under basal or IL-4 stimulation conditions (**Figure 6D**). In IL-33-overexpressing macrophages, in contrast to ST2^−/−^ macrophages, the ratio of phosphorylated p70s6k to p70s6k was higher, and p70s6k activation was significantly lower after Rap treatment (**Figure 6E**). Overall, the data suggest that the IL-33/ST2 axis affects macrophage mitophagy by regulating mTOR activity, thereby leading to metabolic reprogramming and polarization changes.

### IL-33/ST2 signaling in macrophages promotes tumor growth in mice

To investigate the role of the IL-33/ST2 axis in macrophages and its influence on tumor growth, we randomly injected B16 cells into the backs of WT, ST2^−/−^, and IL-33-overexpressing mice. Compared with those in the WT mice, the average tumor weight (*P* < 0.05) (**Figure 6G**), volume (*P* < 0.05) (**Figure 6H**), and body weight (*P* < 0.05) (**Figure 6I**) in ST2^−/−^ mice were lower, but the opposite effects were observed in the IL-33-overexpression group (*P* < 0.05) (**Figure 6K–6M**). These findings demonstrated that a lack of IL-33/ST2 signaling in macrophages inhibits tumor growth (**Figure 6F**), whereas enhanced signaling accelerates growth (**Figure 6J**).

## Discussion

**Figure 7:**
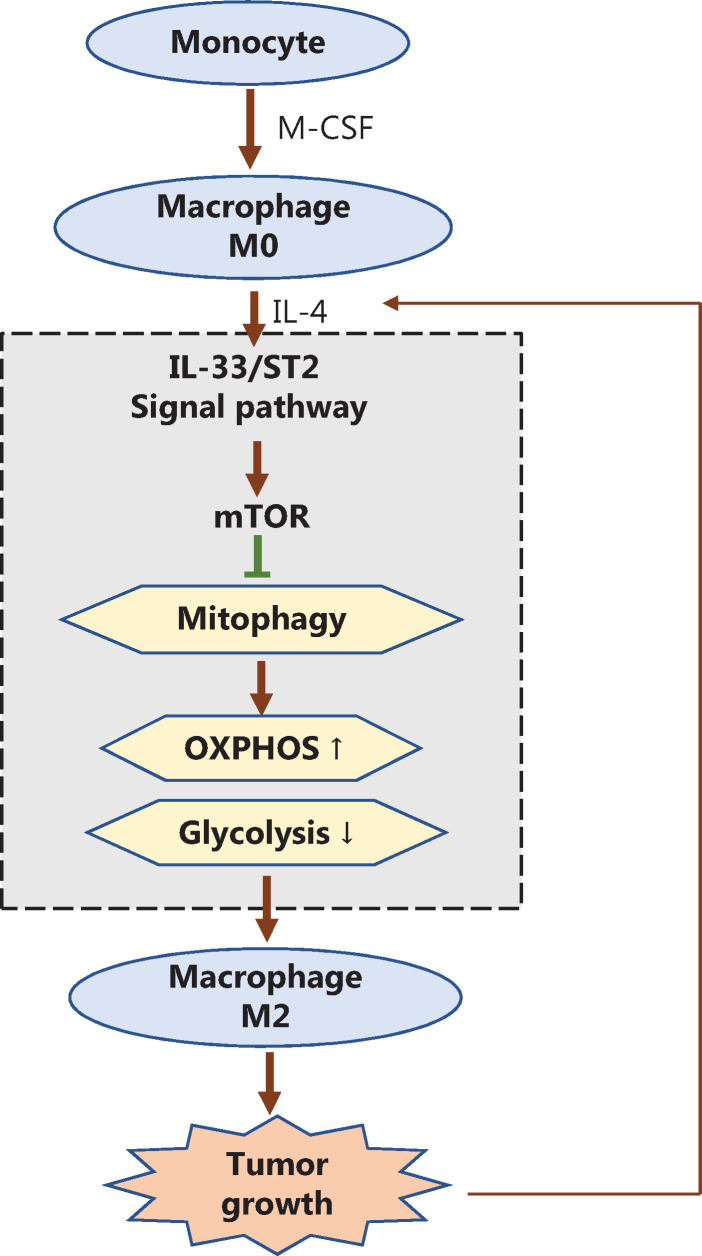
Schematic diagram of how the IL-33/ST2 axis affects tumor growth in the microenvironment by regulating mitophagy of macrophages, thus reshaping their polarization. During the polarization of M2 macrophages induced by IL-4, the IL-33/ST2 axis in differentiated macrophages stimulated by M-CSF inhibits mitophagy by promoting the activity of mTOR, thereby weakening cellular glycolysis. Cellular oxidative phosphorylation is further enhanced, so that the M2 polarization of macrophages is further increased and ultimately promotes tumor growth. In addition, with further tumor development, the microenvironment continues to recruit IL-4 secreting immune cells (such as Th2 and mast cells), which further promote the transformation of macrophages to tumor-promoting M2 through the IL-33/ST2 axis; positive feedback promotes the continuous growth of tumors.

After stimulation from the environment, the adaptive metabolic state of macrophages is essential for their polarization and functionality under physiological and pathological conditions^30^. IL-33/ST2 signaling plays an important role in macrophage polarization; however, the underlying mechanism of action remains unclear^25,31^. Our results suggest that the IL-33/ST2 pathway enhances the M2 polarization of macrophages through reshaping macrophage metabolism by regulating mitophagy (**Figure 7**).

The role of the IL-33/ST2 pathway in cells is complex, because it both protects against certain diseases and amplifies the negative effects of inflammation^32^. Therefore, regulation of IL-33/ST2 signaling may represent a possible target for immune function regulation. Clinically, IL-33 and ST2 receptors are associated with the occurrence and development of autoimmune, allergic, cardiovascular, and neurodegenerative diseases^19,32–36^. For instance, the levels of soluble ST2 protein and IL-33 mRNA in the serum are significantly elevated in people with asthma^37^ but are not associated with the pathogenesis of chronic urticaria^31^. IL-33 is also elevated in infections with Leishmania Donovania, Toxoplasma gondii, and some nematodes^38–41^. IL-33/ST2 signaling pathway-mediated microglia transition from the proinflammatory M1 state to the anti-inflammatory and the tissue-repairing M2 state might be an effective treatment strategy for ischemic stroke^35,42^. In addition, strategies targeting the IL-33/ST2 signaling pathway show promising prospects for the treatment of tumors^43^. Cancer-associated fibroblasts produce high levels of IL-33, which act on TAMs and induce their transition from M1 to M2. Genomic analysis of transition-associated genes in IL-33-stimulated TAMs has shown a 200-fold increase in MMP9 expression^44^. Yang and colleagues have illustrated the interaction mechanism between perivascular cells and TAMs and their ability to promote metastasis through an IL-33/ST2-dependent pathway in a tumor-xenograft mouse model^45^.

However, data from patients with melanoma show some contradictory results: higher IL-33 levels are associated with better survival rates within 80 months (**Supplementary Figure S1**). This finding might be explained by the pleiotropic effects of IL-33; under different microenvironments IL-33 can enhance anti-tumor M1 macrophage polarization^27^. Therefore, although targeting IL-33/ST2 is a potential treatment for a variety of diseases, including cancers, greater understanding of the mechanisms of the IL-33/ST2 pathway in macrophage polarization is required before clinical applications can be considered.

Macrophage activation and polarization are essential for the immune response and monitoring^2,46^. The IL-33/ST2 pathway plays an important role in different types of macrophage polarization. After LPS stimulation, IL-33 upregulates the ability of M1 macrophages to secrete TNF-α, IL-6, IL-1β, and certain chemokines^47^. In addition, breast cancer studies in mice have demonstrated that the IL-33/ST2 pathway leads to the release of cytokines, such as IL-4, IL-5, and IL-13, and promotes polarization of M2 macrophages^24^. These results are also consistent with our findings. Our previous study has demonstrated that deletion of ST2 delays the response to LPS by enhancing the mitochondrial functions of macrophages. WT macrophages downregulate PGC1α and consequently limit mitochondrial proliferation, thereby promoting glycolysis over OXPHOS^27^. However, few studies have investigated the role of the IL-33/ST2 pathway in M2 polarization. This study focused on the metabolic regulators of macrophage polarization and demonstrated that mitophagy plays an important role in M2 phenotype regulation through IL-33/ST2 signaling.

M1 (classical) and M2 (alternative) polarization states have long been paradigms for studying macrophage activation^1,2^. During microbial infection, LPS triggers M1 activation, which is characterized by increased production of proinflammatory and anti-microbial cytokines. M2 macrophages coordinate type 2 immunity by upregulating fibrosis and tissue repair. This type of immunity is activated by stimuli such as IL-4 and IL-13, which are present during parasitic infection^1,48^. These different macrophage functions are probably supported by different metabolic programs^49^. M1 macrophages upregulate glycolytic metabolism, thereby allowing for rapid production of ATP, which might be required during infections with rapid replicating microorganisms. IL-4 polarized M2-like macrophages have similar metabolic characteristics to non-polarized macrophages, with enhanced oxidative respiration, thus increasing energy efficiency (i.e., more ATP production) and therefore compatibility with host defenses against slow-growing and endemic parasites^2^. The recruitment of monocytes is a major event in tumor development. These cells are detected in early primary and secondary human lesions^50^. The phenotype of TAMs is affected by the microenvironment in developing tumors. Environmental factors promote M2 polarization. Therefore, understanding the mechanism of metabolic regulation of M2 polarization is crucial for tumor treatment. Previous studies investigating the metabolic regulation of M2 polarization have shown upregulation of transcriptional induction of the IL-4-mediated nuclear receptors PPAR-°C and PPAR-δ and their coactivator PGC1β, and increased β-oxidation^51,52^. In macrophages lacking PPAR-γ, PPAR-δ, and PGC1β, which are major regulators of fatty acid oxidation and mitochondrial biogenesis, IL-4 suppresses β-oxidation and M2 marker gene expression^10^. A recent study has shown that Myc is upregulated by IL-4 stimulation and controls M2 activation^45^. Generally, Myc is considered a key regulator of oxidative metabolism and other metabolic processes; however, its role in macrophage activation remains unclear and requires further research^53,54^. Our study also focused on the effects of macrophage metabolism on polarization and identified the role of mitophagy in the polarization regulation processes.

Here, we investigated the macrophage metabolic changes under basal and IL-4-stimulated conditions in the absence of ST2 and under overexpression of IL-33. We found that the IL-33/ST2 pathway plays an important role in the metabolic conversion of macrophages from OXPHOS to glycolysis (Warburg effect) by changing the level of mitophagy. We also demonstrated that this metabolic reprogramming is not due to mitochondrial damage, because MMP was not significantly altered by IL-4 stimulation or ST2 knockout, but might be associated with mTOR activity. These results provide a better understanding of the interaction between IL-33/ST2 and macrophage metabolism, and might provide new targets for immunotherapy treatment.
