# Supplementary material for: The IL-33/ST2 axis affects tumor growth by regulating mitophagy in macrophages and reprogramming their polarization
Source: Cancer Biol Med. 2021 Feb 15;18(1):172–83. doi: 10.20892/j.issn.2095-3941.2020.0211 (PMC7877183; doi:10.20892/j.issn.2095-3941.2020.0211)
Supplement: Supplementary file 1 [file cbm-18-172-s001.pdf]

Supplementary material

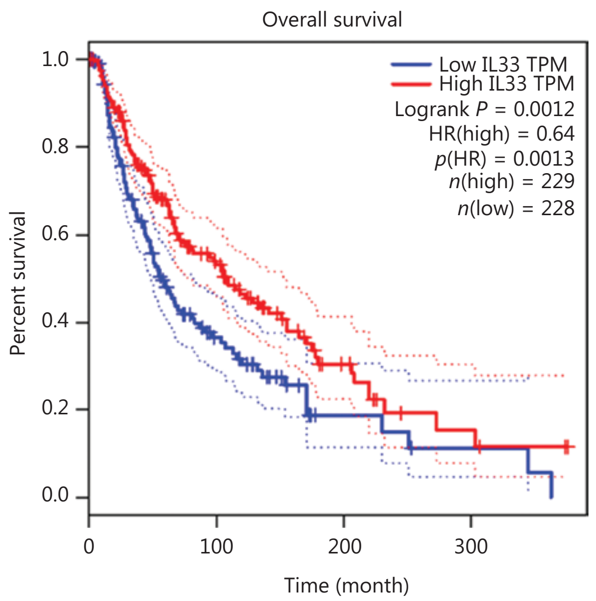

Figure S1 Overall survival curve showing the association between IL-33 and survival in melanoma ($P < 0.05$) acquired from GEPIA. The dotted lines represent the hazard ratio.
